# Assessing a clinical vital sign in severe mental illness: validation study of the 5 sit-to-stand test for monitoring muscle strength – The PsychiActive Project

**DOI:** 10.1192/bjo.2024.842

**Published:** 2025-01-27

**Authors:** Alvaro Lopez-Moral, Diego Munguia-Izquierdo, Javier Bueno-Antequera

**Affiliations:** Physical Performance and Sports Research Centre, Universidad Pablo de Olavide, Seville, Spain; Department of Physical Education and Sport, Universidad de Sevilla, Seville, Spain

**Keywords:** Fitness, early detection, functional test, evaluation, physical health

## Abstract

**Background:**

In individuals with severe mental illness (SMI), low muscle strength heightens the risk of mortality and chronic disease development. Routine muscle strength assessments could identify vulnerabilities, thereby reducing the growing burden associated with SMI. However, integration into clinical settings faces obstacles because of limited resources and inadequate healthcare staff training. The 5 sit-to-stand (5-STS) test offers an alternative for measuring muscle strength compared with more complex or demanding tests. Nevertheless, its validity in individuals with SMI remains unexplored.

**Aims:**

This study aimed to analyse the criterion validity of the 5-STS test in SMI, considering potential age, gender and body mass index influences.

**Method:**

In a cross-sectional study following the ‘STrengthening the Reporting of OBservational studies in Epidemiology’ (STROBE) guidelines, 82 adults with SMI (aged 18–65, 24 women) were assessed. Participants underwent both the 5-STS test and the isometric knee extension strength (KES) test.

**Results:**

Analysis revealed a significant moderate correlation coefficient and intraclass correlation coefficient (−0.58 for both) for all participants, indicating that the measures are valid and assess related aspects of the same construct. Strong agreement was observed in women and the older age groups. The 5-STS test demonstrated accuracy, with a standard error of estimate lower than the within-subject variability on the KES test. Bland–Altman plots showed limits of agreement values of −3.39 and 3.52 for the entire sample, and heteroscedasticity analyses indicated consistent differences between the 5-STS and KES tests across all groups analysed, except in the women's group.

**Conclusions:**

The 5-STS test seems to be a valid test for assessing muscle strength in individuals with SMI, supporting its usefulness for routine assessment in clinical settings, facilitating detection and intervention in critical situations.

Physical fitness emerges as a vital sign and a powerful predictor of disability and mortality in all populations.^1,2^ Cardiorespiratory fitness (CRF) and muscle strength have been the subjects of a great deal of research, showing an additive and multiplicative effect on all-cause mortality.^3^ In addition to CRF, the importance of muscle strength has been increasingly recognised in the onset and development of chronic diseases. Compared with other cofactors such as age, body fat, smoking, alcohol consumption and hypertension, muscle strength has been inversely and independently associated with the risk of all-cause death and cancer in both healthy and clinical populations.^4–6^ Additionally, muscle strength has a protective effect because it is inversely and independently related to the prevalence and incidence of metabolic syndrome,^7,8^ the risk of hypertension in men with previously elevated blood pressure,^9^ and age-related weight gain and adiposity.^10^

People with severe mental illness (SMI), such as schizophrenia, bipolar disorder, schizoaffective disorder or major depression, have lower levels of muscle strength than healthy individuals.^11,12^ Those with major depression experienced a 42% reduction in isometric knee extension strength,^13^ while people with schizophrenia showed a 13% decrease in dynamic leg strength in women and 19% in men,^14^ compared with healthy individuals of the same age and gender. Also, people with schizophrenia exhibited a 25% reduction in muscle power for men and a 30% reduction for women compared with a healthy population in Nygård et al.^14^ Compared with the general population, people with an SMI are two to three times more likely to die prematurely,^15^ reducing their life expectancy by up to 25 years.^16^ The global economic burden associated with SMI was comparable to that of cardiovascular diseases and higher than that of cancer, chronic respiratory diseases and diabetes in 2010, and is estimated to double by 2030,^17^ suggesting that improving the prevention and treatment of SMI is a major health priority.^18^

Routine assessments of muscle strength enable the identification of vulnerable situations and the monitoring of changes in a key health variable, thereby contributing to the mitigation of the increasing burden associated with SMI. However, healthcare staff often overlook the use of these assessments in clinical settings, particularly in low-income countries, because of resource constraints, time limitations and inadequate training. Physical fitness tests represent a reliable and valid option, yet validated muscle strength tests for SMI are currently lacking.^19^ The 5 sit-to-stand (5-STS) test stands out as an efficient assessment of a muscle strength indicator within no more than 10 s, using minimal resources such as a chair, stopwatch and rater, which could address this deficiency. It has demonstrated widespread applicability in both healthy^20^ and clinical populations,^21,22^ providing a faster alternative to more complex or demanding tests because of its lesser reliance on participants’ aerobic resistance, such as the 10-repetition test or the 30-s test. Although several studies have assessed the validity of the test against a reference method for measuring muscle strength,^20–24^ none have been conducted in individuals with SMI, where factors such as balance, sensorimotor and psychological factors (anxiety, depression or vitality) could compromise this criterion.^25^ Furthermore, these studies did not assess concordance between methods but only the strength of the linear association, which could compromise their criterion validity and interchangeability.^26,27^ Therefore, further studies are needed to assess the criterion validity of the 5-STS test, especially in people with SMI, by examining the agreement between the test and the reference methods.

The primary aim of this study was to assess the criterion validity of the 5-STS test in people with SMI and examine the possible influence of age, gender and body mass index (BMI) on the validity of the test. The 5-STS test is hypothesised to have moderate criterion validity for estimating muscle strength in individuals with SMI, regardless of age, gender or BMI. The 5-STS test has been shown to have moderate criterion validity irrespective of age in a previous study with healthy subjects.^20^

## Method

### Study design and participants

From March 2018 to July 2022, a convenience sample of adults diagnosed with psychotic disorders, including schizophrenia, schizoaffective disorder, bipolar disorder, major depressive disorder, personality disorders and severe stress-related disorders, based on ICD-10 criteria and stabilised on antipsychotic medication, was recruited from seven out-patient mental health clinics in southern Spain. Exclusion criteria included clinical instability, comorbid substance abuse or evidence of uncontrolled cardiovascular, neuromuscular and endocrine disorders. Participants underwent anthropometric and physical measurements during a single 30-min session, all administered by a single evaluator trained as an exercise physiologist with over 6 years of experience. The tests were conducted in a consistent order, starting with anthropometric measurements, followed by the 5-STS test and concluding with the knee extension strength (KES) test, which was considered a maximal effort evaluation requiring participants to exert their full strength. Age, diagnosis and duration of illness were obtained from participants’ medical records. Psychiatric symptoms over the previous week were assessed using the Brief Symptom Inventory-18,^28^ with scores ranging from 0 to 72; higher scores reflect greater severity.

The authors assert that all procedures contributing to this work comply with the ethical standards of the relevant national and institutional committees on human experimentation and with the Helsinki Declaration of 1975, as revised in 2013. All procedures involving human subjects/patients were approved by the ethics committees of the Virgen Macarena and Virgen del Rocío University hospitals (1674-N-17) and followed the ‘STrengthening the Reporting of OBservational studies in Epidemiology’ (STROBE) guidance.

Consent for publication was obtained from all individual participants included in the study. Participants were informed about the nature, purpose and potential implications of the research, and they provided written consent, acknowledging their understanding and agreement to the publication of the findings. They did not receive compensation for their participation.

### Anthropometric measurement

Height was measured using a stadiometer (Seca 711, Hamburg, Germany) and body weight via an InBody 770 device (Biospace, Seoul, South Korea). Each measurement was conducted twice, and the average of the two readings was calculated for analysis. The formula for calculating BMI was: body mass (kg) divided by height squared (m²). Measurements were conducted after a 3-h fasting period and without fluid intake, under resting conditions. The participants were instructed to remove all metal objects, wear minimal clothing and urinate before the measurement, which took place in a ventilated examination room under controlled temperature and humidity conditions.

### Measurement of the 5-STS test

The 5-STS test^29^ was conducted with a standardised chair height (0.43 m) without armrests and leaning against a wall. Participants adopted an initial mid-chair sitting position with their backs straight, feet shoulder-width apart and arms crossed over the chest, with palms facing inward. Following ‘ready, set, go!’, the participants performed five repetitions as quickly as possible from a sitting position with buttocks touching the chair to a standing position with full extension. The 5-STS test ended when the participants sat on the chair after the fifth repetition. The time required to complete the task was recorded with a stopwatch, and full extension of the knees in a straight back position was considered a valid repetition. A pre-demonstration was performed, and the participants performed a couple of repetitions for familiarisation. The evaluator stood next to the participant to check for correct execution and verbally encourage the participant. Participants made two attempts with an adequate rest period (120 s), and the best score of the two was used for the analysis.

### Measurement of isometric KES

Isometric KES was measured using a Lafayette Manual Muscle Testing System Model-01165 hand-held dynamometer (Lafayette Instrument Company, Indiana, USA),^30^ which demonstrated a very strong correlation with gold standard methods (1 repetition maximum, *r* = 0.82), suggesting its usefulness in clinical settings.^31^ The protocol used by Mentiplay et al^32^ was replicated, and the participants were placed in a seated position with their hips and knees flexed at 90° and restrained with hands on either side of the stretcher. The dynamometer was placed on the anterior aspect of the tibia proximal to the ankle joint. To minimise the intervention of the evaluator in the recording, the stretcher was placed close to a wall, allowing the evaluator to support their arm and counteract the force produced by the participant. Three attempts for each leg were performed alternately, each lasting 5 s, with a 60-s rest period between attempts. At each attempt, the evaluator verbally encouraged the participants. Following the methodology of previous validity studies,^20,22^ the best body weight-adjusted result (newtons/kg) of all attempts was used for the analysis.

### Data analysis

Participants with a complete and valid data-set of the anthropometric variables, KES and 5-STS tests were included in the analysis. An independent t-test was conducted to assess gender differences in sociodemographic characteristics. Agreement between methods was studied using the intraclass correlation coefficient (ICC),^33,34^ calculated using a two-way mixed model consistency, including the 95% confidence interval. The ICC was interpreted as excellent (0.90 or higher), good (0.75 to 0.90), moderate (0.50 to 0.75) or poor (<0.50).^35^ The standard error of estimate (SEE) was used to quantify the precision of individual test scores. The Pearson's correlation coefficient (*r*) was used as additional information for comparison with existing validity studies of the 5-STS test. According to Mukaka,^36^ a correlation coefficient between 0.3 to 0.5 was considered low, 0.5 to 0.7 moderate and 0.7 to 0.9 high. Agreement between methods was assessed using Bland–Altman plots,^26^ including 95% limits of agreement (LOA). The association between the difference and magnitude of measurement (i.e. heteroscedasticity) was examined using regression analysis. Because of the difference in units between the two methods, gender-specific standardised *z*-scores were calculated for all analyses, which were replicated, accounting for variables such as gender, BMI and age, employing a median-splitting procedure. The data were analysed using the Statistical Package for the Social Sciences (SPSS) (IBM version 25) with a significance level of *P* < 0.05.

## Results

Table 1 presents descriptive data from 82 participants, compared by gender (58 males, 24 females), with an average age of 39.9 years (±9.5), ranging from 18 to 65 years. Significant differences between genders were observed only for height (*P* < 0.001). Predominant diagnoses include schizophrenia, schizotypal and delusional disorders (66%), followed by mood disorders (18%). The 5-STS test, lasting 3–10 s, was performed accurately and without discomfort by all participants.

**Table 1:** Sociodemographic characteristics

Characteristics	All (*n* = 82)	Men (*n* = 58)	Women (*n* = 24)	*P*-value
Age (years)	39.9 ± 9.5 [18–65]	38.6 ± 8.7 [18–60]	43.0 ± 10.6 [20–65]	0.056
Height (cm)	172.5 ± 8.5	176.2 ± 6.2	163.7 ± 6.4	<0.001
Weight (kg)	92.1 ± 21.4	95.2 ± 18.9	84.5 ± 25.2	0.081
Body mass index (kg/m²)	30.8 ± 6.3	30.6 ± 5.5	31.3 ± 8.0	0.659
Illness duration (years)	14.3 ± 9.5	12.8 ± 9.5	16.85 ± 9.2	0.228
Severity of psychiatric symptoms (0–72)^a^	15.5 ± 11.7	14.3 ± 10.6	18.5 ± 13.7	0.157
Diagnoses^b^				
Schizophrenia, schizotypal and delusional disorders	54 (66%)	43 (74%)	11 (46%)	
Mood (affective) disorders	15 (18%)	8 (14%)	7 (29%)	
Neurotic, stress-related and somatoform disorders	3 (4%)	1 (4%)	2 (8%)	
Disorders of adult personality and behaviour	10 (12%)	6 (10%)	4 (17%)	

Values expressed as mean ± s.d., frequency (%) and range [min–max].

a. Severity of psychiatric symptoms was assessed using the Spanish version of the Brief Symptoms Inventory-18.

b. Diagnoses consistent with the ICD-10 categories for severe mental illness.

Table 2 presents the concordance data between the 5-STS and KES tests. Moderate Pearson's correlation coefficient and ICC values were obtained (−0.58 for both) for the whole sample, with a significant correlation between the 5-STS and KES tests (*P* < 0.001). The highest correlation and agreement were observed in the women's group (*r* = −0.70) and in the older age group (*r* = −0.68), and significant correlations between the methods were observed for all the groups (*P* < 0.05). The SEE of the 5-STS test was found to be lower than the intrasubject variability (s.d.) of the KES test.

**Table 2:** Agreement between the 5-STS and KES tests

		5-STS		KES		KES/body weight						
Characteristics	*N*	Mean	s.d.	Mean	s.d.	Mean	s.d.	ICC	95% CI ICC	SEE	*r*	*P*-value
All	82	5.6	1.6	409.4	143.5	4.5	1.5	−0.58	(−0.71, −0.42)	129.5	−0.58	<0.001
Gender												
Men	58	5.2	1.2	460.6	127.3	4.9	1.4	−0.42	(−0.61 −0.19)	122.5	−0.43	0.001
Women	24	6.4	2.0	285.6	98.6	3.5	1.2	−0.63	(−0.82, −0.32)	93.0	−0.70	<0.001
Age (years)												
18–40	42	5.1	1.2	449.7	113.3	4.9	1.2	−0.31	(−0.56, −0.01)	114.2	−0.31	0.049
41–65	40	6.1	1.7	367.1	160.2	4.1	1.6	−0.68	(−0.82, −0.47)	136.7	−0.68	<0.001
BMI category												
Non-obese (BMI < 30)	42	5.8	1.7	443.1	162.2	4.2	1.5	−0.60	(−0.76, −0.36)	132.5	−0.60	<0.001
Obese (BMI ≥ 30)	40	5.3	1.4	373.9	112.0	4.9	1.4	−0.52	(−0.71, −0.25)	106.5	−0.52	0.001

5-STS, 5 sit-to-stand test (time in seconds); KES, knee extension strength (Newtons); BMI, body mass index (kg/m²); ICC, intraclass correlation coefficient; SEE, standard error of estimate.

**Fig. 1:**
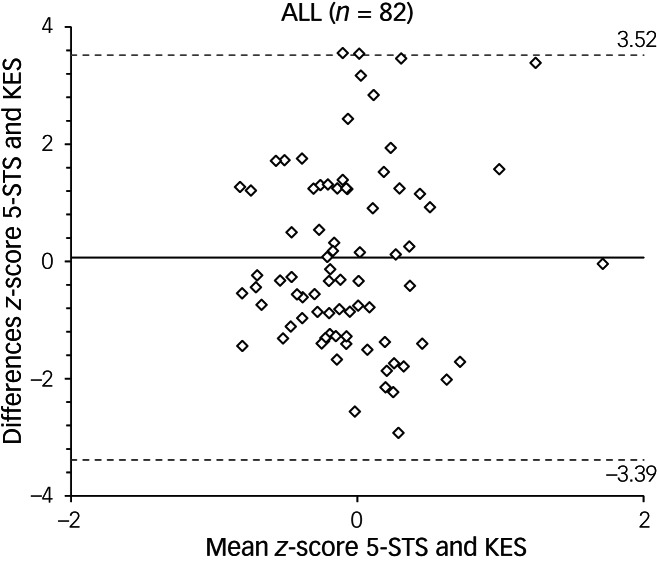
Bland–Altman plots with limits of agreement (LOA) of *z*-scores for the 5 sit-to-stand (5-STS) and knee extension strength (KES) tests in adults with severe mental illness. The thick middle line represents the mean difference between the *z*-scores of the 5-STS and KES tests, while the dashed lines represent the upper and lower 95% limits of agreement (mean difference ± 1.96 s.d.).

Figure 1 illustrates the Bland–Altman plots and the LOA values ranging from −3.39 to 3.52 for the entire sample. No statistically significant differences were detected between the 5-STS and KES methods across all force levels (β = 0.010; *P* = 0.720), indicating homoscedasticity. The narrowest LOA values were identified in the 18–40 age group, ranging from −2.99 to 2.01 (Supplementary Figure 2 available at https://doi.org/10.1192/bjo.2024.842). Differences between methods also remained consistent across all subgroups analysed (β = −0.059 to 0.030; *P* = 0.163 to 1.00), except for the women's group, where differences between methods increased at high levels of assessed force (β = 0.131; *P* = 0.005).

## Discussion

The 5-STS test presented moderate validity for assessing muscle strength in individuals with SMI, with the highest correlation and agreement observed in the women's and in the older age groups. Analyses of the Bland–Altman and heteroscedasticity plots indicated that, regardless of the magnitude of the strength assessed by the 5-STS test, the differences between the 5-STS and KES tests did not increase, an aspect that reinforces interchangeability. Furthermore, our results suggest that the 5-STS test is accurate for estimating muscle strength, as the SEE of the test was found to be lower than the intrasubject variability (s.d.) of the reference test. These findings, coupled with the heterogeneity of the sample and the analysis of other factors such as age, gender and BMI, provide further scientific evidence for the utility of the 5-STS test in routine assessment of muscle strength in clinical settings involving SMI.

The criterion validity of the 5-STS test in individuals with SMI had not been studied before. Therefore, we discuss our findings in comparison with evidence from other populations where the validity of the 5-STS test had been explored to evaluate lower body strength. In adults aged 55–65 years with chronic stroke, a correlation of −0.68 was observed for affected knee extensors and −0.48 for unaffected knee extensors, as measured with a hand-held dynamometer in relation to the 5-STS test.^24^ The correlation with the affected knee extensors closely mirrored the correlation observed in our study for the older age group (*r* = −0.68), a factor that reinforces our findings and supports the criterion validity of the 5-STS test for other clinical populations within the same age range. However, variations in correlations between affected and unaffected knee extensions suggest that muscle weakness in an affected leg is a limiting factor influencing the performance of the 5-STS test, leading to a decrease in the correlation between methods. Despite the moderate correlation, methodological limitations, like not normalising strength values to body weight and concerns about the quality of the 5-STS execution test because of an emphasis on speed, may limit the validity of the 5-STS test.

In individuals with multiple sclerosis, two studies provided insights into the correlation between the 5-STS and KES tests. Özüdoğru et al^23^ observed correlations of −0.87 for the left leg and −0.81 for the right leg when comparing these measures in a group of 23 adults, using a hand-held dynamometer (Lafayette). Additionally, Møller et al^22^ reported a correlation of −0.71 for the most affected leg and a correlation of −0.30 for the least affected leg in a group of 11 adults using an isokinetic dynamometer. The high correlations reported in Özüdoğru et al^23^ and Møller et al^22^ could be attributed to substantial muscle deterioration in individuals with multiple sclerosis. Özüdoğru et al^23^ demonstrated mean strength values of 42.21 *N* for the right leg, 44.00 *N* for the left leg and an average time of 16 s for the 5-STS test. Conversely, Møller et al^22^ indicated mean strength values of 165.7 Nm for the most affected leg, 180.4 Nm for the least affected leg and an average time of 9 s for the 5-STS test. In contrast, our study showcased notably higher mean strength values of 409.4 *N* and a shorter average time of 5.6 s for the 5-STS test. Additionally, as discussed earlier, Møller et al^22^ aligned with the findings of Mong et al,^24^ indicating stronger correlations in the more affected leg compared with the less affected leg, implying that muscle weakness in the more affected leg serves as a limiting factor influencing the 5-STS test. These findings underscore the potential impact of muscle degradation on the observed correlations in individuals with multiple sclerosis. Nevertheless, despite these discrepancies, the correlation rates persisted at a moderate to high level, consistently aligning with our study, which reinforces the criterion validity of the 5-STS test for individuals with SMI.

Complementarily, Jones et al^21^ reported a correlation of −0.38 between maximal isometric quadriceps contraction, measured by a strain gauge, and the 5-STS test in adults aged 59–79 years with chronic obstructive pulmonary disease. The correlation obtained in this study was considerably lower compared with the correlation observed in Mong et al^24^ and our study for the older age group, which was −0.68 for both. The main difference between the studies which could explain these disparities lies in the measurement instruments used as reference. Jones et al^21^ used a strain gauge, whereas a hand-held portable dynamometer was utilised in our study and in Mong et al's study.^24^ While both strain gauges and hand-held dynamometers can be used for muscle strength assessment, hand-held dynamometers generally offer higher accuracy because of their direct measurement of muscle force, rigorous calibration procedures and standardised measurement techniques. Strain gauges, conversely, rely on indirect measures of muscle force, which may introduce additional sources of error and variability in measurement accuracy. Factors such as calibration play a critical role in ensuring the accuracy of strain gauge measurements.^37^ These factors not only influence the accuracy of strain gauge measurements but also highlight potential discrepancies between different strain gauge models, which could significantly impact the interchangeability and correlation of measurements obtained with this instrument.^38^

Finally, across the entire sample, our study obtained a correlation coefficient slightly lower than Bohannon et al^20^ (−0.58 versus −0.63) for individuals aged 14–85 years. The methodologies of both studies were similar, as they both compared the 5-STS test with the KES test measured by a portable dynamometer (MicroFET), which could explain the similar results. Nevertheless, Bohannon et al^20^ included a diverse range of participants, including both young individuals (<18 years) and elderly subjects (>65 years), in contrast to our study, which focused exclusively on adults (18–65 years). The inclusion of these diverse age groups may explain the observed variations in correlation results, adding complexity to the determination of criterion validity for a specific age-related subset of the population. In addition, our study performed the KES test with participants seated on a stretcher, with their hands held at their sides, and the hand-held dynamometer was stabilised by the assessor's arm resting on a wall. In Bohannon et al,^20^ the participants were seated stabilised with straps, and the hand-held dynamometer was secured with a belt. Stabilising and securing the dynamometer to an external element reduces interference and evaluator error in the measurements, whereas stabilising the position of the participants during the test may affect muscle activation and, consequently, the force values recorded.^39^ The presence of variations in stabilisation methods and participant conditions may be a contributing factor in Bohannon et al,^20^ who achieved a slightly higher correlation between tests. However, despite these differences, the correlation between the 5-STS and KES tests remained moderate. The use of stabilisation methods may be a barrier to implementing the 5-STS test in clinical settings, especially with SMI, limiting its feasibility in epidemiological or large-scale investigations.

The criterion validity of the 5-STS test has been extensively studied across diverse populations. Despite inherent variations, its consistent demonstration of a moderate relationship with muscle strength, as measured by hand-held dynamometry, underscores its robustness. The observed correlations parallel the results of our study, supporting the validity of the 5-STS test for individuals with SMI. Nevertheless, future research should adhere to standardised measurement protocols to avoid methodological biases and facilitate result-comparison across studies. Additionally, utilising the ICC parameter is recommended for assessing criterion validity.

### Limitations

One of the main limitations of this study was the use of a predominantly male sample of adults with SMI, potentially constraining the interpretation of validity results. However, despite subgroup data presentation, the higher prevalence of males limits the generalisability of findings to males. Furthermore, we observed that female participants were, on average, older and had spent more time in the mental health system compared with their male counterparts. However, statistical analyses indicated that these differences in age and duration of illness were not statistically significant. Additionally, differences in the specificity of the 5-STS test, which assesses muscle strength in dynamic movements, and the KES test, which evaluates maximal force in a fixed position, could constrain the results. Therefore, it is recommended that the criterion test follows a similar movement pattern and velocity to the 5-STS test.^40^ Furthermore, we did not consider the influence of other factors, such as balance, sensorimotor abilities and psychological factors like anxiety, depression or vitality, which could affect 5-STS test performance.^25^ Various participant conditions that could influence muscle strength, such as muscle or joint pain, previous experience and motivation, were not recorded. These aspects could limit the agreement between the two methods and should be considered in future studies.

Despite these limitations, this study incorporated measures of concordance, correlation and SEE in the statistical analysis, which enhanced the interpretation of results and addressed some of the limitations seen in previous studies. Importantly, this study was the first to assess the validity of the 5-STS test in adults with SMI, contributing to the reduction of the growing burden associated with this population by facilitating routine screening of muscle strength in clinical settings as a vital sign. In addition, a larger sample size was used compared with previous studies on the criterion validity of fitness tests in this population.^19^ Additionally, a subgroup analysis explored potential variation factors that could affect criterion validity, thereby enhancing the generalisability of the results to the broader clinical population. Finally, a standardised measurement protocol was applied for the KES test, and all the tests were performed by a single experienced evaluator to ensure standardisation and minimise interferences during the process.

### Relevance for clinical practice

The 5-STS test, with proven validity, accuracy and practicality, stands outs as a rapid, secure and easily accessible alternative to assess muscle strength, in contrast to more demanding tests, like the 30-s test. It also serves as a key indicator of physical functioning, which is associated with independence and quality of life, and its simplicity facilitates its use by behavioural health providers. Incorporating the 5-STS test into clinical settings, especially where resources are limited or staff are untrained, can significantly enhance patient care by enabling early detection of muscle impairments and risk situations that require urgent intervention.

## Supporting information

Lopez-Moral et al. supplementary material Lopez-Moral et al. supplementary material

## Data Availability

The data-sets generated and/or analysed during the current study are available from the corresponding author on reasonable request.
