# Supplementary material for: Assessing a clinical vital sign in severe mental illness: validation study of the 5 sit-to-stand test for monitoring muscle strength – The PsychiActive Project
Source: BJPsych Open. 2025 Jan 27;11(1):e24. doi: 10.1192/bjo.2024.842 (PMC11823003; doi:10.1192/bjo.2024.842)
Supplement: Lopez-Moral et al. supplementary material [file S2056472424008421sup001.docx]

**(SUPPLEMENTARY) FIGURE 2**

*Bland-Altman Plots with limits of agreement (LOA) of* z*-scores for the 5-STS and KES tests in adults with SMDs*

**A**

*Note.* Results are stratified by sex (A: men, B: women), age (C: 18–40 years, D: 40–65 years) and BMI (E: < 30 kg/m², F: ≥ 30 kg/m²). The thick middle line represents the mean difference between the *z*-scores of the 5-STS and KES tests, while the dashed lines represent the upper and lower 95% LOA (mean difference ± 1.96 *SD*).

**B**

**C**

**D**

**E**

**F**
